# In Search of the Holy Grail: A Specific Diagnostic Test for Rheumatic Fever

**DOI:** 10.3389/fcvm.2021.674805

**Published:** 2021-05-14

**Authors:** David J. McMillan, Rukshan A. M. Rafeek, Robert E. Norton, Michael F. Good, Kadaba S. Sriprakash, Natkunam Ketheesan

**Affiliations:** ^1^School of Science and Technology, Engineering and Genecology Research Centre, University of the Sunshine Coast, Maroochydore, QLD, Australia; ^2^School of Science and Technology, University of New England, Armidale, NSW, Australia; ^3^Pathology Queensland, Townsville University Hospital, Douglas, QLD, Australia; ^4^Faculty of Medicine, University of Queensland, Brisbane, QLD, Australia; ^5^Laboratory of Vaccines for the Developing World, Institute for Glycomics, Griffith University, Gold Coast, QLD, Australia; ^6^Queensland Institute of Medical Research Berghofer (QIMR) Berghofer Medical Research Institute, Brisbane, QLD, Australia

**Keywords:** rheumatic fever, rheumatic heart disease, diagnostic test (MeSH), group A streptococcus, M protein

## Abstract

Current diagnosis of Acute Rheumatic Fever and Rheumatic Heart Disease (ARF/RHD) relies on a battery of clinical observations aided by technologically advanced diagnostic tools and non-specific laboratory tests. The laboratory-based assays fall into two categories: those that (1) detect “evidence of preceding streptococcal infections” (ASOT, anti-DNAse B, isolation of the Group A *Streptococcus* from a throat swab) and (2) those that detect an ongoing inflammatory process (ESR and CRP). These laboratory tests are positive during any streptococcal infection and are non-specific for the diagnosis of ARF/RHD. Over the last few decades, we have accumulated considerable knowledge about streptococcal biology and the immunopathological mechanisms that contribute to the development, progression and exacerbation of ARF/RHD. Although our knowledge is incomplete and many more years will be devoted to understanding the exact molecular and cellular mechanisms involved in the spectrum of clinical manifestations of ARF/RHD, in this commentary we contend that there is sufficient understanding of the disease process that using currently available technologies it is possible to identify pathogen associated peptides and develop a specific test for ARF/RHD. It is our view that with collaboration and sharing of well-characterised serial blood samples from patients with ARF/RHD from different regions, antibody array technology and/or T-cell tetramers could be used to identify streptococcal peptides specific to ARF/RHD. The availability of an appropriate animal model for this uniquely human disease can further facilitate the determination as to whether these peptides are pathognomonic. Identification of such peptides will also facilitate testing of potential anti-streptococcal vaccines for safety and avoid potential candidates that may pre-dispose potential vaccine recipients to adverse outcomes. Such peptides can also be readily incorporated into a universally affordable point of care device for both primary and tertiary care.

“*Despite the increase in knowledge of rheumatic fever, no specific diagnostic test has been forthcoming. This is a distinct deterrent to the advancement…*.” *T. Duckett Jones (1944)* (1)

## Introduction

**Figure 1:**
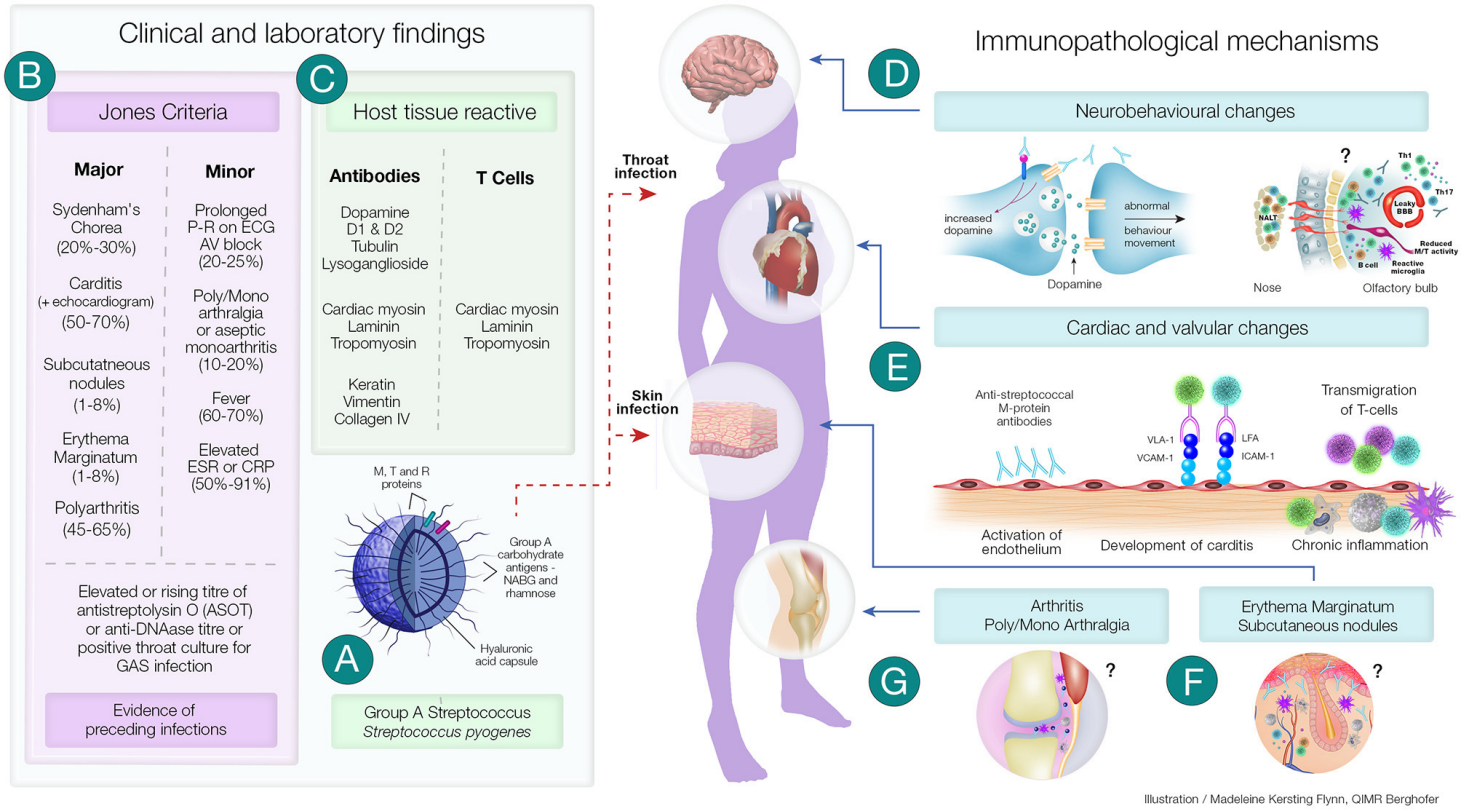
Clinical and Immunopathological Features of Acute Rheumatic Fever and its Complications. **(A)** Group A streptococci (GAS) have several virulence factors that enables it cause a variety of superficial and deep-tissue infections. In a proportion of individuals following pharyngitis or pyoderma, one of the post-streptococcal autoimmune sequalae that can develop is acute rheumatic fever (ARF). **(B)** A group of universally accepted clinical manifestations and non-specific laboratory investigations form the Revised Jones Criteria. Laboratory investigations include evidence of recent streptococcal infection (as assessed by rising titre of anti Streptolysin O (ASOT) or anti-DNase titre or positive throat culture for streptococcal infection). Evidence of preceding streptococcal infection in addition to either two major or one major and two minor criteria confirms the diagnosis of ARF. **(C)** Both clinical and experimental studies have shown the presence of antibodies and CD4^+^ T cells generated in ARF have the ability to cross-react with both streptococcal proteins and host tissue proteins. The autoimmune inflammatory process initiated by these antibodies and T cells lead to several of the immunopathological changes observed in ARF. **(D)** Sydenham's Chorea, a group of neurobehavioral abnormalities observed in ARF has been found to involve anti-dopamine, anti-β-tubulin and anti-lysoganglioside antibodies reacting with D1 and D2 dopamine receptors, signalling kinases and ion channels and causing these abnormalities. Recent studies have also shown that the CD4^+^T cells may be involved in breaching the blood brain barrier and facilitating the entry of antibodies and inflammatory cells. **(E)** *Streptococcus* specific antibodies can upregulate VCAM-1 and ICAM-1 on vascular and valvular endothelial cell. Activation of these cells lead to transmigration of activated streptococci specific T-cells into heart tissue leading to cross-reactive responses with tissue proteins perpetuating inflammatory responses including neovascularisation and the appearance of granulomatous lesions in cardiac tissue. **(F)** Although direct experimental evidence is space, anti-streptococcal antibodies that cross-react with laminin, tropomyosin, vimentin and keratin in the skin may cause the characteristic rash—erythema marginatum observed in ARF. Furthermore, the formation of subcutaneous nodules may be due to delayed hypersensitivity type responses against streptococcal antigens. **(G)** Anti-streptococcal antibodies could also form immune complexes which bind to the synovial membrane and/or collagen in joints leading to inflammation of the synovial tissue causing arthralgia and arthritis. Repetitive streptococcal infections drive the autoimmune process leading to chronic inflammation and carditis, culminating in rheumatic heart disease and if untreated it is followed by congestive cardiac failure and death. ^?^Mechanisms not well-characterised; ICAM-1, Intercellular adhesion molecule-1; LFA, Leukocyte associated function antigen; Jones Criteria, (% of patients presenting with the specific feature); Th1 and Th17 CD4^+^, T cell subsets; VLA-1, Very late antigen-1; VCAM-1, Vascular cell adhesion molecule-1.

In 2005, the WHO estimated that annually over 700 million people worldwide suffered from two of the commonest forms of Group A Streptococcal (GAS) infection—GAS pharyngitis and GAS pyoderma. Of the over 500,000 annual deaths due to complications of GAS infections, well over 65% were attributed to rheumatic heart disease (RHD) (2, 3). Acute rheumatic fever (ARF) and RHD, which are autoimmune sequelae of GAS pharyngitis and/or pyoderma, have ceased to be major public health problems in high-income countries. However, in some of these countries it is still highly prevalent among Indigenous populations and occasional outbreaks of ARF/RHD occur among the wider population. In middle and low-income countries, which account for more than 80% of the world's population, poverty, household overcrowding, and poor access to timely medical care continue to be associated with high incidence/prevalence of this disease. Control efforts in these countries continue to be fraught with several confounding factors, namely paucity of accurate data, unavailability of preventive measures such as safe and effective vaccines, non-specific diagnostic tools, and lack of treatment options. The diagnosis of ARF is mostly based on a clinical algorithm initially described in 1944 (1) with later modifications. The current Modified Jones Criteria for diagnosis of ARF take into account as to whether the patient resides in a low, moderate or high -risk population (4) (Figure 1). Although, a specific unequivocal laboratory diagnostic test has been long envisioned and possibly within our reach, it is yet to be realised.

## The Utility of the Jones Criteria for Diagnosis of Rheumatic Fever

Over seven decades have passed since Duckett Jones set forth a well-defined group of “major and minor criteria” for “the diagnosis of rheumatic fever” in his seminal publication (1). This was during the pre-antibiotic era when salicylates were the therapeutic agent of choice for treating ARF. These criteria were intended to be useful “until the aetiology of rheumatic fever is known or there is a specific diagnostic test.” They were developed to avoid confusion and misdiagnosis of acute ARF/RHD and provide a rational basis to develop programs for prevention and patient care. Since then, the additions and modifications made to the original criteria, which now form the “Revised Jones Criteria” (4, 5) still do not prevent misdiagnosis (6–9).

In response to the falling incidence of ARF in the USA, changes were made to improve the specificity of the criteria at the expense of sensitivity. This resulted at times in an underdiagnosis of the disease in high-incidence populations. The consequences of under-diagnosis in these populations, in generally low resource environments, could be considerable and possibly greater than those of over-diagnosis. In 2006, the first version of the Australian Rheumatic Fever Guidelines incorporated additional criteria, and of subclinical carditis, aseptic monoarthritis and polyarthralgia as major manifestations in high-risk groups. Subsequently in 2012, monoarthralgia was included as a minor manifestation. In 2015, the American Heart Association (AHA) further revised the Jones criteria to separate moderate-high and low-risk populations, and to include echocardiography as a tool to diagnose cardiac involvement (4). They noted that the new guidelines aligned more closely with the Australian guidelines and these 2015 re-revised Jones criteria were endorsed by the World Heart Federation. Of the laboratory tests, in addition to a positive throat culture and elevated or rising titre of anti-streptolysin O (ASOT) which were described by Jones we have now added anti-DNase titre. However, these are non-specific laboratory tests that are used to determine an exposure to streptococcal infection and are of little use in the definitive diagnosis of ARF/RHD, particularly in regions where streptococcal infection is endemic (6–9). Therefore, a robust specific diagnostic test that can be used in the laboratory setting is required to overcome misdiagnosis of ARF/RHD. We need to acknowledge that the scientific information that has been accumulating over the last 75 years on the molecular and cellular mechanisms involved in the pathogenesis of the disease process have not translated to the development of a specific low-cost diagnostic test for ARF/RHD. Such a test could significantly contribute to the accuracy of ARF/RHD diagnosis, in particular in regions where ARF/RHD is still rampant. Indeed, the availability of such a specific diagnostic test could make the Jones Criteria redundant in the diagnosis of ARF/RHD as Duckett Jones himself may have intended.

## Problems With the Current Diagnosis of ARF

In several countries, with increased awareness of hygiene and relatively low-density living conditions, the incidence of ARF has declined. However, for the majority of world populations living in socio-economically deprived conditions, the incidence of ARF is still high (4). Although in some countries ARF is a reportable disease, the data may greatly underestimate the incidence because of uncertainty in diagnosis or misdiagnosis.

Firstly, the Jones criteria, rely on a set of criteria that together are used of clinical diagnostic purposes. Several of these criteria are associated with other conditions and can lead to misdiagnosis. Another problem lies in the accepted association of GAS infection, solely in the setting of pharyngitis with ARF. Thirdly a lack of a specific diagnostic laboratory marker for ARF further hinders early confirmation of the diagnosis. Several epidemiological observations suggest that in some populations, skin infections may also pre-dispose to ARF (10). Furthermore, *Streptococcus dysgalactiae* subspecies *equisimilis* (SDSE) may also be an aetiological agent for ARF (11). Indeed, in many humid regions skin infection, and SDSE throat isolation rates are common. In such regions, the GAS isolation rates from throat swabs do not correlate with the reported high incidences of ARF (12, 13). Moreover, many subjects carry GAS and SDSE in their throat, which often could erroneously contribute to high throat isolation rates of these bacteria and may not have contributed to the pathogenesis of ARF. Notwithstanding the above difficulties, most clinicians adhere to the traditional revised Jones criteria to diagnose ARF.

Understanding ARF pathogenic mechanisms may help in identifying a suitable diagnostic marker for ARF. Unfortunately, GAS is a human-specific pathogen and ARF manifests only in humans. Fortunately, however, recent work using the Lewis Rat model for RHD is changing this scenario. Histopathological presentation of heart tissues in the rats injected with GAS M5 strain is akin to that observed in RHD patients (14–22). Since ARF precedes RHD, it is reasonable to assume that the RHD-like histological changes in Lewis Rats exposed to GAS may have progressed through stages that exhibit partly analogous manifestations to ARF in humans. One such major manifestation relates to neurobehavioral changes as in Sydenham's Chorea (SC). Indeed, in our recent study (23) we have also demonstrated neurobehavioral changes in rats injected with whole killed GAS. To develop a standalone specific laboratory diagnostic test for ARF/RHD, it is imperative that the early mechanisms leading to the development of the disease process is well-characterised.

## The Aetiopathogenesis of Rheumatic Fever and Its Complications

The post-streptococcal autoimmune process that leads to ARF/RHD is multifactorial. Genetic pre-disposition, the type or strain of streptococcal pathogen, frequency of infection and the site of infection contribute to the disease process. However, the relative importance of each of these factors remains unknown. Although several studies in different regions showed that ARF/RHD may be linked to specific MHC antigens, both Genome Wide Association studies (24) and transcriptome based studies (25) are still in their infancy in ARF/RHD research and yet to provide a clear mechanistic pathway in disease pathogenesis (26).

Streptococcal pharyngitis and skin infections activate humoral and cell-mediated immune responses against streptococcal virulence factors. Some of the antibodies and T cells generated during the infection process cross-react with host tissue proteins, a hallmark of ARF/RHD immunopathogenesis. To develop a specific and low-cost diagnostic that can be used in ARF/RHD endemic countries, it is essential to have a good understanding of the molecular and cellular aetiopathogenesis of rheumatic fever and its complications. While epidemiological and clinical studies on patients with ARF/RHD have contributed to our understanding of the burden of this human-specific disease, decades of microbiological, immunological and animal studies have complemented the clinical finding by enabling hypothesis driven research to verify and understand the mechanisms involved in the multisystem clinical manifestations.

Antibodies generated against GAS M protein and *N*-acetyl-beta-D-glucosamine cross-react with cardiac tissue proteins. It has been demonstrated that monoclonal antibodies against these antigens, derived from patients with ARF (27), cross-react *in vitro* with human myosin and valvular endothelium. Furthermore, injection of recombinant streptococcal M protein induces autoantibody and autoreactive T cell that leads to carditis and valvulitis in the Lewis autoimmune valvulitis model (18). Antibodies and T cells derived from these rats also activate aortic endothelial cells in culture (21) facilitating transmigration of activated T cells across the endothelial barrier. These, observations have added further evidence that cross-reactive antibodies generated against streptococcal proteins that bind to tissue proteins is a major mechanism involved in the immunoinflammatory process observed in ARF/RHD leading to carditis (Figure 1). There is also evidence that structural similarities between tissue proteins, such as laminin and vimentin, could be the basis of antibody-mediated damage to valve structures. T-cell clones derived from valvular lesions from patients react with myosin and valve-derived proteins (28) and T cells from rats injected with GAS M protein release inflammatory cytokines upon exposure to these and streptococcal antigens *in vitro*. The Th1/Th17 inflammatory response may also facilitate epitope spreading within the valvular tissue and further expose other tissue antigens such as vimentin and collagen perpetuating and amplifying the inflammatory process (29). Chronic inflammation leads to characteristic changes observed in cardiac tissue in ARF/RHD including neovascularisation, giant cell formation and fibrosis.

There is considerable evidence that some streptococcal M protein N-terminus domains bind the CB3 region of Collagen type IV and initiate an autoantibody response to collagen establishing an inflammatory process leading to the spectrum of disease presentation observed in ARF/RHD (30). These autoantibodies do not cross-react with streptococcal M proteins (31, 32). The proponents of this concept view that the pathology in ARF/RHD points to the sub-endothelial collagen matrix being the primary site of the inflammatory process due to the systematic targeting of collagen matrix by these antibodies. Due to the distinctive structure of the heart valves and the endothelium, the inflammatory process progresses to valvular scaring. This in turn leads to the haemodynamic changes that progress to RHD. Of the several autoantibodies observed in patients with ARF/RHD (33, 34) some cross-react with streptococcal proteins (“molecular mimicry”) and the others do not (“collagen neoantigen”). Although, the proponents of the “molecular-mimicry” and the “collagen neoantigen” may consider the mechanisms that initiate the disease process to be distinct, it is conceivable that both these mechanisms contribute to the disease process that culminates in host tissue damage.

Antibodies against GAS *N*-acetyl-beta-D-glucosamine cross-react with neuronal cells in the basal ganglia, leading to deposition of immune complexes causing excessive release of dopamine that form the basis of the symptomatology observed in SC (35, 36). Recent studies in mouse models have also shown GAS infection of the olfactory epithelium can cause breaches in the blood brain barrier and facilitate T cell infiltration into the brain (37, 38). However, further work on the development and clinical progression remains to be done. The migratory and transient manifestations observed in joints and the characteristic rash and subcutaneous nodules have been partly attributed to the accumulation of immune complexes that cause these clinical manifestations and are part of the “major” Jones Criteria used in the diagnosis of ARF. However, compared to the pathogenesis of carditis, there is a paucity of experimental data on the pathogenesis of SC, arthritis, erythema marginatum and the development of subcutaneous nodules in ARF.

## Enabling the Design and Develop a Novel Disease Specific Diagnostic Tool

Point-of-care rapid antigen tests are currently available to diagnose GAS infection. While these tests are diagnostic for a current GAS infection, they are not diagnostic for ARF/RHD. It has also been shown that a rise in anti-Streptolysin O titre (ASOT) is less prominent in recurrent ARF than during the first episode (6). Moreover, lab-based detection of serum ASOT or Anti-DNase B (ADB) antibodies used to diagnose a recent streptococcal infection are non-specific and of little value in regions of high streptococcal endemicity. A recent attempt using a Triplex assay combining the ASOT and ADB with anti SpnA antibodies noted that while promising, this only confirms recent GAS infection and not ARF/RHD (39). This highlights the importance of identifying robust markers of ARF/RHD that can be incorporated into point-of-care tests to be used in low-income countries. Such point-of-care tests simplified in a lateral flow assay format can also overcome the limited laboratory facilities available in low-income countries.

The autoimmune nature of ARF indicates that specific peptides present in GAS antigens trigger the host responses that ultimately results in ARF/RHD. Despite decades of research, the identity of the specific epitopes which contribute to ARF/RHD, and which could form the basis of a diagnostic, are yet to be identified. The challenges in identifying these peptides are immense. Above, we outlined how the lack of an animal model until recently has precluded lab-based immunological studies or ARF/RHD. From a host perspective, we do not know if the same epitopes are responsible for ARF/RHD in different patients. Several studies also suggest host susceptibility to ARF/RHD is associated with HLA type (40). As ARF/RHD occurs after a GAS infection, it is also not possible to recover the specific GAS isolates responsible for case of the autoimmune process that triggers ARF.

**Figure 2:**
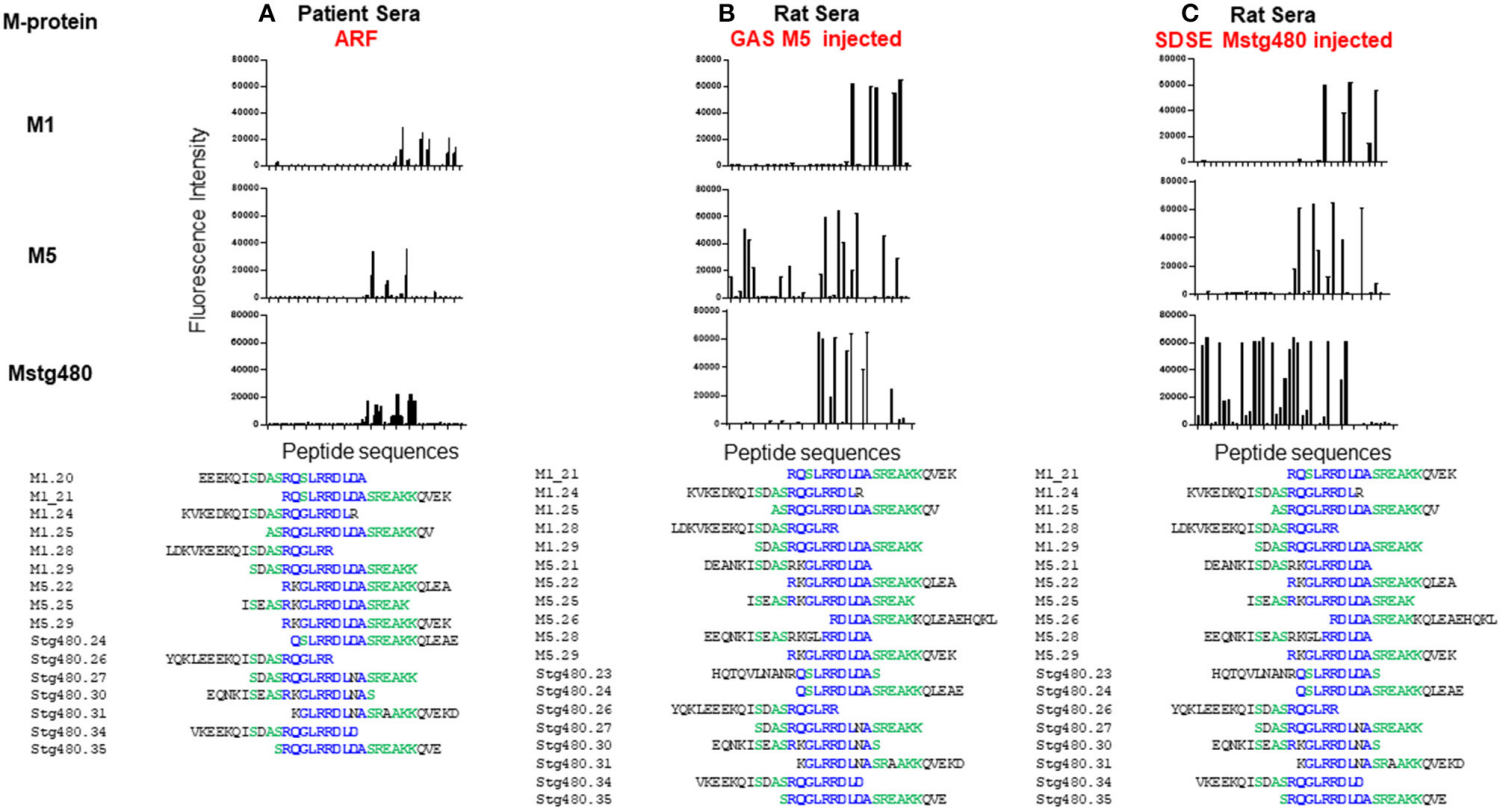
Antibody reactivity with peptides from streptococcal M protein. Fluorescence intensity (RFI) of peptides screened with; **(A)** pooled human sera from patients with ARF (*n* = 7), **(B)** pooled sera from rats following five injections of GAS M5 (*n* = 3), and **(C)** pooled sera from rats following five injections of SDSE Mstg480 (*n* = 3). Rat serum samples were collected 224 days following initial injections. Peptide microarrays consisting of 186 overlapping 20 mer peptides derived from M1, M4, M5, and Stg480 M-proteins were constructed by JPT technologies. Sera samples were diluted in blocking buffer (1:200) and incubated with the peptide array for 1 h at 30°C. The primary sera were removed, arrays washed and incubated with fluorescently labelled anti-human-IgG or anti-rat-IgG antibody at 0.1 μg/ml for 1 h. After washing and drying the microarrays were scanned using a high resolution fluorescence scanner (635 nm) to obtain fluorescence intensity profiles. The y-axis represents the fluorescence intensity (FI) of experimental sample with FI from negative control sera subtracted. The x-axis represents peptides from M-proteins shown on the left Peptides with a FI >5,000 were aligned using Clustal Omega. Amino acids present in >75% of aligned sequences are coloured blue. Peptides present in >50% of aligned sequences are coloured green. (Deidentified pooled human serum samples were collected under ethics approval #HREC/15/QTHS/134 and rat serum under #JCUA2083).

Our group is addressing this deficit through combining our RAV model with advances in peptide array technology. Peptide arrays are a recent advance that enable the systematic screening of thousands of peptides for reactivity with specific sera samples. Using this technology, we have screened pooled sera from patients with ARF and controls for reactivity with 186 20 mer peptides derived from three M proteins. Sixteen peptides, all of which contained all or part of the same conserved sequence motif reacted with the sera (Figure 2). This peptide motif, (ASRQGLRRDLDASREAKKQV; P20) is found in the conserved C-terminal region of all three proteins represented on the array. Control sera were not reactive against these peptides. To establish whether the observed peptide reactivity in human was due to the M protein, we also probed the array with antisera raised against M5 protein generated in our RAV model. We again saw reactivity with the same set of peptides (Figure 2). Subsequent bioinformatic analysis has revealed P20 to be 100% conserved in 72 of 175 different M-types. Moreover, the same conserved motif is present in an M-protein from a SDSE isolate that was isolated from an individual presenting with ARF (11). When these GAS and SDSE M-proteins were injected subcutaneously into separate groups of Lewis rats, antibodies to the same motif were found to be predominant.

The epitope we identified is congruent with the amino acid region used to define Class I and Class II proteins (41), and within various C-repeat regions. Our peptide results are not proof that anti-P20 antibodies contribute to ARF/RHD but suggest that the higher titres may be used as a serological marker of ARF/RHD in some patient groups. Expanding our research to include individual sera samples from well-characterised patient cohorts (including patients with recent streptococcal infection), and expansion of our peptide arrays to represent all the peptides present in the various M-protein types, and indeed all peptides encoded in GAS genome, represents a generational change in the way that antibodies from ARF/RHD patients reactive with GAS epitopes can be identified and used for diagnostic purposes. Peptide arrays also have one other advantage over previous approaches. The screening of one array requires very small amounts of sera (<50 μl). Current ARF/RHD sera collections can therefore be leveraged against these arrays, leaving valuable human sera remains available for other research studies. Well-catalogued serial patient samples from several regions are required to identify the specific peptide/s that would serve as targets for both specific diagnostic development and mechanistic studies. Moreover, if future studies also included collections of DNA samples, variable autoimmune immune responses following GAS infection can be linked to human genetic variation. Given the complexity of ARF/RHD such an exhaustive and comprehensive approach may be the only path to a true specific diagnostic and greater understanding of these diseases.

## How Does Detecting Antibodies to Disease Specific Streptococcal Epitopes be of Value in Developing Vaccines for Streptococcal Infections?

A rat model of myocarditis and valvulitis was developed (14) and it was subsequently demonstrated that immunisation with a pool of 15 peptides from the C-repeat region of the M-protein induced mononuclear cell infiltration into the hearts of Lewis rats (16). A further study, however, found that while peptides from the A-repeat region of the M-protein induced significant myocarditis, peptides from the B-repeat region induced mild carditis and that peptides from the C-repeat region did not induce any carditis (42). Studies in 2016 (43) and 2020 (44) then showed that immunisation of rats with the leading peptide vaccine candidate from the C-repeat region of the M-protein, “J8,” “J14,” and “p^*^17” did not induce cardiomyopathy.

CD4^+^ T-cells are the most common T-cell subset identified in diseased valves (45). The only human T-cell epitopes that have been identified as a possible cause of valvulitis are from the A-repeat region. One hundred and sixty three human T-cell clones were generated from diseased valves and tested against M-protein peptides from the A-repeat region and against heart proteins (46). Twenty percent of the clones recognised M-protein peptides and these only reacted with three A-repeat region peptides. Some also reacted with heart proteins. Since T-cell epitopes from the A-repeat region of the M-protein have been implicated in pathogenesis, the frequency and phenotype of T-cells specific for these epitopes could be assessed using combinatorial tetramer staining. This technology has been used to detect and characterise CD4^+^ T-cell influenza-specific epitopes at frequencies as low as 1 per million in peripheral blood (47). The same technology could identify A-repeat region-specific CD4^+^ T-cells and may provide laboratory diagnosis of some patients with active rheumatic valvulitis.

As stated, with the enormous burden of GAS infection, a streptococcal vaccine is urgently needed. How else can we harness the recent discovery of the ARF/RHD rat model for safe vaccine design? In the sections above we describe identification in the GAS M protein derived peptides that reacted with sera derived from rats injected with recombinant M5 (Figure 2). These peptides are largely similar to or overlap with the peptides that are reactive to ARF human sera (Figure 2). This important observation, namely similarity of reactions between the rat model and patients has implications to further assess safety of M protein-based vaccine candidates. So far, the M-protein based peptide vaccine candidates have been painstakingly designed with the aim of deletion of deleterious T cell epitopes to make the vaccine candidates safe. While the efficacy of such candidates has been amply tested, there has been no direct assessment of these candidates for propensity to cause or exacerbate reactions that could lead to ARF. Additionally, we also need to demonstrate that the human cardiac tissue cross-reactive antibodies in ARF and in the GAS-injected rats react to similar or overlapping peptides of these tissue proteins.

## Conclusion

Since the clinical criteria for diagnosis was first described (1), we have gained significant understanding of the pathogenesis of rheumatic fever and its complications that provide an adequate foundation to develop prototype antibody-based specific diagnostics. It is our view that by exploiting available technologies and having access to serial serum samples of patients with rheumatic fever from different regions coupled with studies on the rat autoimmune valvulitis model, it is possible to test and identify a specific antibody-based assay to simplify the diagnosis of rheumatic fever. The COVID-19 pandemic has demonstrated that rapid detection tools, basic infection control measures, international information and resource sharing can provide a platform to adequately mitigate an infection regardless of the economic status of individual nations. While it remains to be seen whether developing a specific and low-cost diagnostic test is achievable by the centenary of the first publication of the diagnostic criteria for rheumatic fever, the pursuit of the holy grail may rely on the proposition that “simplicity is the ultimate sophistication.”^1^

## Data Availability Statement

The raw data supporting the conclusions of this article will be made available by the authors, without undue reservation.

## Ethics Statement

The studies involving human participants were reviewed and approved by #HREC/15/QTHS/134. Written informed consent to participate in this study was provided by the participants' legal guardian/next of kin. The animal study was reviewed and approved by #JCUA2083.

## Author Contributions

All authors were involved in the conceptualisation of the content of the manuscript and contributed equally in preparing and editing the manuscript.

## Conflict of Interest

The authors declare that the research was conducted in the absence of any commercial or financial relationships that could be construed as a potential conflict of interest.

## Footnotes

^1^Aphorism attributed to the renaissance polymath Leonardo da Vinci (1452–1519).
